# Exploring Spanish writing abilities of children with developmental language disorder in expository texts

**DOI:** 10.3389/fpsyg.2024.1360245

**Published:** 2024-04-11

**Authors:** Raquel Balboa-Castells, Nadia Ahufinger, Mònica Sanz-Torrent, Llorenç Andreu

**Affiliations:** ^1^NeuroDevelop eHealth Lab, eHealth Center, Universitat Oberta de Catalunya (UOC), Barcelona, Spain; ^2^Estudis de Psicologia i Ciències de l’Educació, Universitat Oberta de Catalunya (UOC), Barcelona, Spain; ^3^Departament de Cognició Desenvolupament i Psicologia de l’Educació, Secció Cognició, Universitat de Barcelona (UB), Barcelona, Spain; ^4^Institut de Neurociències, Universitat de Barcelona (UB), Barcelona, Spain

**Keywords:** developmental language disorder (DLD), specific language impairment (SLI), writing abilities, shallow language, expository text

## Abstract

**Introduction:**

Numerous studies have shown that children with developmental language disorder (DLD), in addition to oral language difficulties, exhibit impaired writing abilities. Their texts contain problems in grammar, organization, cohesion, and length of written output. However, most of these studies have been conducted with English speakers. English is characterized by complex phonological structure, opaque orthography, poor morphology and strict word order. The aim of this research is to observe the writing abilities of children with DLD in a language with simple phonological structure, transparent orthography, rich morphology and flexible word order like Spanish in the production of expository texts.

**Methods:**

Twenty-six children with DLD (mean age in months = 128.85) and 26 age-and sex-matched typically developing (TD) children (mean age in months = 124.61) wrote an expository text about their favorite animal.

**Results:**

In order to analyze how the two groups plan and encode written texts, we looked at word frequency and sentence structure, grammatical complexity and lexical density, and omissions and errors. Compared to the TD group, the children with DLD omitted more content words; made more errors with functional words, verb conjugation and inflectional morphemes, and made a large number of spelling errors. Moreover, they wrote fewer words, fewer sentences, and less structurally and lexically complex texts.

**Discussion:**

These results show that children with DLD who speak a transparent orthography language such as Spanish also have difficulties in most language areas when producing written texts. Our findings should be considered when planning and designing interventions.

## 1 Introduction

Developmental language disorder (DLD)—also known as specific language impairment (SLI)—is a neurodevelopmental disorder affecting around 7.5% of the child population (Tomblin et al., 1997; Norbury et al., 2016) with no significant difference in sex distribution (Calder et al., 2022). DLD is defined as a severe and persistent disorder in oral language acquisition and development, unassociated with a medical condition, such as hearing loss, intellectual disability, autism, or any neurological disorder or genetic syndromes (Bishop et al., 2016). Moreover, DLD may co-exist with other neurodevelopmental disorders such as attention-deficit, hyperactivity, motor, speech and behavioral problems, or dyslexia (Bishop et al., 2016).

Studies of children with DLD have found that they exhibit an heterogeneous oral language profile (Conti-Ramsden, 2008) which may, to differing degrees, involve one or several expressive and receptive language components and which affects social and/or school development (e.g., Leonard, 1998; Bishop et al., 2017). Previous studies have documented significant difficulties across the different components of oral language including phonology, morphology, syntax, vocabulary, semantics, pragmatics, discourse, and verbal learning and memory (Bishop et al., 2017). Research have found some phonological issues in productive phonology such as omissions of unstressed syllables and final consonants and changing syllabic structures (e.g., omitting final consonants and reducing consonant and vowel clusters; Aguilar-Mediavilla et al., 2002, 2007; Bishop and Clarkson, 2003; Gallon et al., 2007; Broc et al., 2013; Larkin et al., 2013). Additionally, children with DLD struggle with other phonological abilities, such as in phonological awareness abilities including complex tasks like deleting phonemes, substituting phonemes, or producing rhyming words (Thatcher, 2010; Vukovic et al., 2022; Korlaet et al., 2023). A considerable body of research has focused on studying morphological problems in children with DLD. They have deficits in the use of inflectional morphology, such as verb tense and agreement. Specifically, they omit the ending-s in the third singular person (e.g., ‘She read a book’ instead of ‘She reads a book’) and the past tense marker-ed (e.g., ‘Yesterday, I play with Rachel’ instead of ‘Yesterday, I played with Rachel; Van der Lely and Ullman, 2001; Abel et al., 2015). Research has shown that these children struggle also with functional words since they omit articles, pronouns and prepositions (Bedore and Leonard, 2001; Restrepo and Gutierrez-Clellen, 2001; Sanz-Torrent et al., 2007; Coloma et al., 2016). Children with DLD produce syntactically simpler sentences (Marinellie, 2004) and find it difficult to understand both complex syntactic structures, such as dependent clauses and passive sentences (e.g., Bishop, 1997; Leonard and Deevy, 2006; Novogrodsky and Friedmann, 2006; Montgomery and Evans, 2009; Van der Lely et al., 2011; Leonard, 2014). Different studies have analyzed vocabulary and semantics in children with DLD and have observed that they typically present smaller and less rich lexicons than their typical peers (McGregor et al., 2023) and show slower latency times and more errors in picture naming (Lahey and Edwards, 1996; Lahey and Edwards, 1999; McGregor et al., 2002). Moreover, in receptive single-word vocabulary tests, they tend to score within the average range but statistically lower than their matched TD peers (Gray et al., 1999; McGregor et al., 2002, 2013; Sheng and McGregor, 2010; Haebig et al., 2015). Furthermore, they show semantic impairments encompass problems with expressing or understanding meaning from word combinations (Katsos et al., 2011). In pragmatics, children with DLD have difficulties understanding figurative language such as metaphors, double meanings or idiomatic expressions (Norbury, 2005) and understanding communicative intentions (Andrés-Roqueta and Katsos, 2020).

Having these problems in oral language, children with DLD are very likely to also experience difficulties in literacy. Different studies have reported that they are at greater risk for reading difficulties than children with typical language development (TD) (Sanz-Torrent et al., 2010; Bishop et al., 2017; Adolf and Hogan, 2018). It has been estimated that around 50% of children with DLD present also dyslexia (Bishop et al., 2009; Ramus et al., 2013) and they may exhibit reading comprehension difficulties (Ramus et al., 2013; Gough Kenyon et al., 2018).

Regarding writing, according to Hayes and Flower (1980) model, the writing process can be divided into three stages: planning, translating, and revising. The planning stage involves generating ideas, organizing thoughts and ideas, and setting writing goals. The translation stage is where these ideas are transformed from oral language into written form, and the revising stage involves reading and editing the written material. Additionally, the Not-So-Simple View of Writing (Berninger et al., 2002; Berninger and Amtmann, 2003) which is a modification of the Hayes and Flower model that incorporates various cognitive and executive function components, including working memory, into the writing process. Thus, children with DLD are expected to face difficulties in producing written content due to their challenges both in oral language and executive functioning, particularly in working memory (Im-Bolter et al., 2006; Archibald and Joanisse, 2009; Montgomery et al., 2010; Ebert and Kohnert, 2011). Accordingly, different studies have found problems in written production in children with DLD (i.e., Broc et al., 2021; Tucci and Choi, 2023). Written productions can be evaluated accordingly through analyses of microstructure and macrostructure (Liles et al., 1995). According to Hughes et al. (1997), microstructure refers to the syntactic and lexical levels of the production, that is, the language form and content. It has been characterized in terms of productivity as well as complexity. Conversely, macrostructure denotes the hierarchical structure and coherence of the text beyond the level of a single sentence. The way the story’s episodes are arranged, how events are sequenced, and how the protagonists’ internal states drive or respond to the story’s events are all examples of macrostructure (e.g., McCabe and Peterson, 1984; McCabe and Rollins, 1994; Liles et al., 1995).

As far as we know, previous studies about writing in children with DLD have focused on analyzing the microstructure. Tucci and Choi (2023) performed a scoping review of literature focused on the effects of DLD on writing skills across the lifespan. Results showed that spelling may be the most vulnerable area for individuals with DLD. In this sense, previously studies show that children with DLD make a significantly higher percentage of spelling errors when producing written texts compared to TD children of the same age (Mackie et al., 2013; Williams et al., 2013; Dockrell et al., 2014; Reilly et al., 2014; Joye et al., 2019). For the most part, these spelling errors are due to phonological and morphological errors, which involve substituting, inserting or eliminating letters within a word (Mackie and Dockrell, 2004). Broc et al. (2021) conducted a scoping review about the nature of spelling errors in children with DLD across different orthographies. They divided the 18 reviewed studies into those based on dictation tasks and those containing written narratives because these two types of tasks involve different writing processes. In addition, they separately analyzed those two types of studies regarding the typology of the orthography of language in which they were carried out (opaque or transparent orthographic system). On dictation tasks, children with DLD produced more phonologically unacceptable spelling errors. These errors varied by age and by the nature of the words dictated. Moreover, children with DLD produced less phonologically unacceptable spelling errors when the spelling could be derived by applying one-to-one sound-letter correspondences (transparent orthographic system) than when the phoneme-grapheme correspondences were irregular (opaque orthographic system). On written narratives, they found that most of the studies to assess the spelling skills of children with DLD had been conducted in opaque orthographies and only identified one study conducted in a transparent orthography language, Spanish (Soriano-Ferrer and Contreras-González, 2012). Overall, on written narrative tasks, results were contradictory about phonologically unacceptable spelling errors. Some studies found more difficulties than the control groups (Mackie and Dockrell, 2004) but others did not replicate those results (Broc et al., 2013; Dockrell and Connelly, 2015). In the only study conducted in a transparent orthography (Soriano-Ferrer and Contreras-González, 2012) children with DLD produced more spelling errors that were phonologically unacceptable compared to their peers of the same age. However, both groups made four times as many errors when the phoneme correspondence was irregular, as opposed to when it was regular, resulting in observable errors. Finally, on written narratives, only studies conducted in opaque orthographies were reported to examine errors in inflectional morphological spelling. Children with DLD overall showed problems in their ability to accurately use inflections in their spelling. Error patterns in children with DLD were similar to younger language matched peers but more frequent than their age-matched peers.

Moreover, children and adolescents with DLD also may have difficulties with grammar, organization, cohesion, and length of written output (Tucci and Choi, 2023). They make more errors and omissions when writing nominal inflectional morphemes and using derivational morphemes (prefixes and suffixes) than age-matched children (Connelly et al., 2011; Mackie et al., 2013). For example, they produce more errors in the use of plural forms and past simple verb tenses (Windsor et al., 2000; Mackie and Dockrell, 2004; Larkin et al., 2013). Additionally, they use fewer words that contain prefixes, such as *im/patient* or *dis/agree* and suffixes, such as *teach/er* or *profession/al* (Mackie et al., 2013). Interestingly, these differences are still significant when children with DLD are matched with children possessing the same language skills. The percentage of omitted auxiliary verb *be* and content words—such as nouns as subjects—is also significantly higher than in TD children of the same age with a similar receptive vocabulary (Windsor et al., 2000; Mackie and Dockrell, 2004). Moreover, they display poorer lexical diversity (Scott and Windsor, 2000; Mackie et al., 2013; Williams et al., 2013; Levlin and Waldmann, 2020; Stuart et al., 2020). The differences are once again significant when children with DLD are compared to a younger cohort with the same language skills. Research has also shown differences in writing abilities to be significant when children with DLD are compared to younger children of a similar reading age (Mackie et al., 2013). In addition, written texts produced by children with DLD are also shorter (i.e., contain fewer written words; Mackie et al., 2013; Dockrell et al., 2014; Stuart et al., 2020; Ralli et al., 2021) and contain a lower percentage of meaningful syntactic units (T-units) and coordinated sentences than texts produced by TD children of the same age. The sentences in their texts are also significantly shorter and contain fewer words per clause (Scott and Windsor, 2000; Mackie et al., 2013).

Most of these studies about the characteristics of the written language in DLD have been conducted with English speakers, a language with an opaque orthographic system. However, English has a number of characteristics that make it very different from transparent orthographic system languages such as Spanish. First, Spanish has a simple phonological structure. It has approximately 23 phonemes and the majority of syllables follow a simple consonant–vowel (CV) structure and have limited clusters and blends (Gorman and Gillam, 2003; Goikoetxea, 2005). Therefore, in Spanish, segmentation of syllables at the level of onset–rime is often equivalent to segmentation at the phonemic level. For example, a Spanish speaker who segments the syllables of the word “casa” [house] into onset and rime (onset:“c,” rime: “a”) will also identify the phonemes that make up the word (/c/ /a/ /s/ /a/; Goswami, 2008). This is not the case for the English, where many onsets and rimes contain clusters of phonemes, as in “sport” and “cost” which must be segmented further (De Cara and Goswami, 2002). Spanish has a transparent orthography both in terms of reading and writing since practically every phoneme is represented by a single, unique letter. Thus, children need to learn fewer phoneme-to-letter conversions in Spanish than in English, where one phoneme can be represented by multiple spellings (as the phoneme /f/ in words like frog, tough, and photo). Moreover, Spanish is a morphologically rich language: it uses inflections to indicate the relationship between the elements; that is, the composition of the words changes (e.g., “casa” [house], “casas” [houses], “casita” [little house]). This implies important morphological changes in words that include a lexeme or radical morpheme, to which one or more grammatical morphemes can be added (e.g., cas-a, cas-as, cas-ita). Another feature of Spanish, as in other Romance languages, is that the order of the words within the sentence is flexible (e.g., “Juan ama a Maria” [“John loves Mary”]; “A Maria ama Juan” [“To Mary loves John”]; “Juan a Maria ama” [“John to Mary loves”]), although there is a basic order of words in the sentence (canonical order: subject > verb > object). In addition, Spanish is a pro-drop language that allows personal pronouns to be dropped in the sentence (“Juega al fútbol” [Plays football]).

Previous cross-linguistic research suggests that both the simple phonological structure of Spanish and its highly regular phoneme-to-letter correspondence facilitate the process of learning to read and write in Spanish children with TD (Müller and Brady, 2001; Seymour et al., 2003; Ellis et al., 2004; Ziegler et al., 2010; Florit and Cain, 2011; Castejón et al., 2015). In theory, this should also help Spanish-speaking children with DLD as they learn to write. However, to our knowledge only two studies have been conducted concerning written language production among the DLD population in Spanish (Soriano-Ferrer and Contreras-González, 2012; Buil-Legaz et al., 2023). Buil-Legaz et al. (2023) analyzed the difficulties of children with DLD in spelling. Participants were 18 children with DLD (aged 7;0–11;5, M = 8;4, SD = 1.25) and 18 children with DLD with TD (aged 7;0–11;6, M = 8;2, SD = 1.29) that completed a spelling-to-dictation task of words and pseudowords, where length was manipulated. They used digital tablets to collect data and obtain measures of accuracy, latencies and total writing durations. Results showed that children with DLD produced more errors, longer latencies and longer writing durations than age-matched children. Regarding accuracy, analysis of the errors showed that children in the control group produce few errors, most being substitutions, while children with DLD made more errors and of more varied types (substitutions, omissions and additions). Moreover, they were more affected by length on writing accuracy than the control group. Soriano-Ferrer and Contreras-González (2012) examined the written narrations and the influence of linguistic measures on narrative competence of children with DLD. Children did a written narrative task, where they had to recall, in writing, a story given to them previously orally twice. The story was composed of 19 propositions, with a simple grammatical structure. The children with DLD created short narratives, poorly organized and less cohesive. Also, their writings contained more syntax errors and had a higher proportion of phonologically inaccurate spelling errors in natural spelling but not in arbitrary spelling errors.

However, in these two previous studies, written performance was measured by single-word dictation (Buil-Legaz et al., 2023) and by a narrative based on a story given orally (Soriano-Ferrer and Contreras-González, 2012). As stated Broc et al. (2021), in dictated tasks the words to be written are predetermined while on written narratives participants can choose words they know, which may result in fewer spelling errors as they may opt for words they feel to spell. However, in the study of Soriano-Ferrer and Contreras-González (2012) children had to retell in writing a story given orally. Therefore, as far as we know, there are not any studies that analyze the production of expository texts in Spanish-speaking children with DLD. An expository text or informational text is a non-fiction text that gives facts and information about a topic. It aims to inform, explain, describe, or define a particular topic or subject. Its primary purpose is to present factual information, clarify ideas, and provide insights in a clear, concise, and organized manner. This kind of text is very common in subjects such as science, history and social sciences. There are several different types of expository structures. Meyer and Ray (2011) proposed six structures: compare-and-contrast, problem-and- solution, cause-and- effect, sequence, enumeration, and description. Each text structure type represents a distinct text organization and purpose. For example, description focuses on describing a topic, person, place or thing by listing a collection of its features or examples while. Expository texts serve as valuable tools for assessing writing abilities in children due to their inherent structure, which demands clear, organized, and coherent expression of ideas. Evaluating a child’s ability to comprehend, synthesize, and communicate information effectively can be achieved through their creation of expository texts (Fisher and Frey, 2017). In this way, the aim of this study was to examine and compare text writing of children with DLD in the production of expository texts in Spanish. More specifically, by looking at how these children and their aged-matched TD counterparts plan and encode expository texts, we sought to find out what variables are most frequently impaired in children with DLD compared to the TD group. We hypothesize that, given the errors that children with DLD tend to make in oral language, we expect to find significantly more difficulties in this population compared to TD children in all areas of writing. Specifically, we expect to find more inflectional morpheme and verb conjugation errors, as well as a higher percentage of omitted functional words.

## 2 Materials and methods

This study was approved by the Universitat Oberta de Catalunya’s (UOC) Ethics Committee. Furthermore, it was conducted in accordance with the ethical standards laid out in the 1964 Declaration of Helsinki and subsequent updates (WMA. World Medical Association, 2013).

### 2.1 Participants

This sample of children was a subsample of the study conducted by Ahufinger et al. (2021), which included 70 children (35 children with DLD and 35 typically developing (TD) children). The subset of children in this study included 52 participants (12 girls and 40 boys): 26 children had DLD (mean age in months = 128.85 (10.73 years); SD = 25.02; range: 95 to 188 months) and 26 were age-and sex-matched TD children (mean age in months = 124.61 (10.38 years); SD = 24.25; range: 90 to 184 months). All participants met the following inclusion criteria: (a) nonverbal intellectual quotient (NVIQ) > 75; (b) typical hearing at 500, 1000, 2000 and 4,000 Hz at 20 dB, in accordance with the American National Standard Institute (1997); (c) typical or corrected vision; (d) typical oral and speech motor abilities, as confirmed by a certified speech language pathologist; and (e) native Spanish-Catalan bilingual speakers as reported by parents. Children were excluded if parents reported (a) other biomedical conditions commonly linked to genetic or neurological causes, such as autism, intellectual disability, Down syndrome or Williams syndrome (Bishop et al., 2017); (b) frank neurological signs; or (c) seizure disorders or the use of medication to control seizures.

In 2017, children with DLD were identified with the help of the Catalan Center of Resources for Language-and Hearing-Impaired People (CREDA), members of the Catalan service for school counseling and guidance (EAP), and the Catalan Association of Specific Language Impairment (ATELCA), who work in conjunction with public and private schools throughout Catalonia to identify children with DLD or children with language difficulties. Children in the DLD group had a formal diagnosis of DLD or a suspected diagnosis and were in the process of being diagnosed, or were children whose families or teachers were concerned about language difficulties and/or were receiving speech–language services at the time of the original study per parental report. The TD children were recruited from public schools within the larger Barcelona metropolitan area. The TD children were at grade level in school, had no history, or diagnosis, of language learning disability, and had never received speech and language services.

To confirm each participant’s language status, standardized tests were administered by two trained researchers at the time of the study. These were the non-verbal IQ (NVIQ) Kaufman Brief Intelligence Test (K-BIT; Kaufman and Kaufman, 2004) and the Clinical Evaluation of Language Fundamentals - Fourth Edition, Spanish (CELF-4 Spanish; Semel et al., 2006). In the latter case, the researchers evaluated and recorded the participants’ Core Language Score, Expressive Language Score and/or Receptive Language Score. For the children with DLD, the CELF Core, Expressive or Receptive composite scores were at least 1.5 standard deviation below age-level expectations. For the children in the TD group, the CELF composite scores were all at or above age-level expectations. Each child with DLD was matched with a TD child of the same sex and age (+/− 3 months), as shown in Table 1.

**Table 1:** Age and standardized cognition and language measurement scores for the group of children with developmental language disorder (DLD) and the group of typical developing (TD) children.

	DLD (*n* = 26)			TD (*n* = 26)			Comparison	
Variable	Mean	SD	Range	Mean	SD	Range	t(50)	*p*
*Age in months*	128.85	25.02	95–188	124.61	24.25	90–184	0.62	*p* = 0.54
K-BIT mat (NVIQ)^a^	98.96	11.57	77-119	102.85	9.84	88–129	−1.30	*p* = 0.20
CELF-CLS^b^	73.50	10.62	45–89	108.58	8.32	95–130	−13.63	***p* < *0*.01**
CELF-ELS^c^	73.65	8.61	52–85	107.92	9.25	89–128	−13.82	***p* < *0*.01**
CELF-RLS^d^	78.27	10.05	59–97	104.77	7.98	90–122	−10.53	***p* < *0*.01**

For each variable, the scores by age have a mean of 100 and a standard deviation (SD) of 15 (except age in months). Significance *p*-values are shown in bold. NVIQ, non-verbal intelligence quotient.

a K-BIT mat = Kaufman Brief Intelligence Test, Spanish version: non-verbal intelligence quotient score (Kaufman and Kaufman, 2004).

b CELF-4 CLS = Spanish Clinical Evaluation of Language Fundamentals, Fourth Edition: Core Language Score (Semel et al., 2006).

c CELF-4 ELS = Spanish Clinical Evaluation of Language Fundamentals, Fourth Edition: Expressive Language Score (Semel et al., 2006).

d CELF-4 RLS = Spanish Clinical Evaluation of Language Fundamentals, Fourth Edition: Receptive Language Score (Semel et al., 2006).

Table 1 shows no significant age or NVIQ differences between the participants with DLD and their matched TD peers when the sample was selected. However, the children with DLD obtained significantly lower scores than the TD group on the three CELF language test scales.

In 2019, these children were invited to participate in the current study to examine and compare text writing abilities in the production of expository texts. All families were asked to sign a new consent form following the IRB protocol from the Universitat Oberta de Catalunya (UOC).

### 2.2 Instruments and procedure

Children completed two testing sessions of approximately 60 min each. These sessions were part of a larger study examining reading and writing skills. In the first session, children completed a reading assessment. In the second session, children completed two brief oral morphological tasks first, and the writing test for the present study after. The time allocated to carry out the writing task was approximately 15/20 min per child. The evaluation sessions were carried out individually in the research laboratories of the Universitat Oberta de Catalunya and the Universitat de Barcelona by two research assistants trained for this purpose. All participants were administered the narration writing task from the Spanish Batería de Evaluación de los Procesos de Escritura (Writing Process Evaluation Battery – PROESC; Cuetos et al., 2018). The children had to write an expository text about their favorite animal. If they were unable to think of an animal, they were given suggestions such as cats, dogs or lions. The children were given unlimited time to write a one-page text explaining whatever they wanted to about the animal. The instructions on how to complete the task were given in Spanish, but the examiner explained to the children that they could write the text in the language they preferred. All the participants wrote the text in Spanish.

### 2.3 Coding

The children’s texts were transcribed for analysis using the Codes for the Human Analysis of Transcripts (CHAT) program and were analyzed using the Child Language Data Exchange System (CHILDES) Project’s Computerized Language Analysis (CLAN) program (MacWhinney, 2000). The following category system was created to study the transcribed data, drawing on Mackie et al. (2013) and Salas and Caravolas (2019):

#### 2.3.1 Word frequency and sentence structure

- Total number of words (TNW): Total number of words written in the text.
- Number of different words (NDW): This index was used to score the lexical diversity of the vocabulary in the text. To prevent an effect caused by the length of the text, we calculated Guiraud’s R index: types/√tokens (Guiraud, 1954).
- Main clauses ($MC): Total number of simple sentences the child has written as a proportion of the total number of sentences. A simple sentence is defined as a meaning unit that has a noun phrase, functioning as subject, and a verb phrase, functioning as predicate. Thus, it is a sentence expressing a single action, e.g., ‘El gato come verdura’ (‘The cat eats vegetables’).
- Coordinate clauses ($CC): Total number of coordinate sentences the child has written as a proportion of the total number of sentences. A coordinate sentence consists of two simple clauses, with equal syntactic importance, linked by a conjunction, e.g., ‘El perro ladra y el gato maúlla’ (‘The dog barks and the cat meows’).
- Subordinate clauses ($SC): Total number of subordinate sentences the child has written as a proportion of the total number of sentences. A subordinate sentence consists of a simple independent clause and at least one simple dependent clause. In subordinate sentences, dependent clauses do not make sense on their own; they need to be embedded in the independent clause to convey their meaning, e.g., ‘El perro, que es mi animal favorito, come comida de perro’ (‘Dogs, which are my favorite animal, eat dog food’).
- Total number of clauses (TNC): Total number of main, coordinate and subordinate clauses.
- Words per clause (WpC): This index is calculated by dividing the total number of words the child produces by the total number of clauses in the text.

#### 2.3.2 Grammatical complexity and lexical density

- Number of adjectives ($NAj): Number of adjectives the child uses in the text.
- Number of adverbs ($NAv): Number of adverbs the child uses in the text.
- Number of connectors ($CO): Number of connectors the child uses in the text. Connectors include conjunctions, e.g., ‘y’, ‘o’ and ‘también’ (‘and’, ‘or’ and ‘also’), and discourse markers, e.g., ‘Primero de todo’ and ‘finalmente’ (‘first of all’ and ‘finally’). The purpose of linguistic connectors is to provide contextual meaning and clarity to the text by combining sentences and paragraphs.

#### 2.3.3 Errors

- Spelling errors ($SE): Spelling errors in the children’s texts are defined using the categories established by Matute et al. (2010).
- Omission errors ($SEo): Omitting a letter, syllable or segment from the word, e.g., writing ‘hose’ instead of ‘horse’.
- Joining words ($SEw): Omitting the space between words, e.g., writing ‘elcaballo’ (‘thehorse’) instead of ‘el caballo’ (‘the horse’).
- Segmentation errors ($SEs): Dividing words incorrectly, e.g., writing ‘con migo’ (‘to gether’) instead of ‘conmigo’ (‘together’).
- Translocation errors ($SEt): Changing the letter or syllable order in a word, e.g., writing ‘fuetne’ (‘soucre’) instead of ‘fuente (‘source’).
- Addition errors ($SEa): Adding a letter or syllable to a word, e.g., writing ‘cominida’ (‘dininer’) instead of ‘comida’ (‘dinner’).
- Phoneme substitution errors ($SEp): Substituting an unvoiced sound for a voiced sound, e.g., ‘peso’ (‘weight’) instead of ‘beso’ (‘kiss’). An English example would be ‘pear’ instead of ‘bear’.
- Articulatory substitution errors ($SEas): Natural spelling errors caused by substituting a consonant for another that has a close production point, e.g., ‘cato’ instead of ‘gato’ (‘cat’), and/or a similar mode of articulation, e.g., ‘mida’ instead of ‘mira’ (‘look’). An English example would be ‘coal’ instead of ‘goal’ for the first case and ‘deal’ instead of ‘real’ for the second.
- Arbitrary spelling errors ($SEar): Spelling errors related to spelling rules. In Spanish, these manifest as substitution errors between the letters /v,b/, /c,s,z/, /g,j/, /y,ll/ and /h,∅/., e.g., ‘cantava’ instead of ‘cantaba’ (‘sang’). An English example would be ‘liv’ instead of ‘live’ or ‘werked’ instead of ‘worked’.
- Accent errors ($SEc): Errors such as ‘tenia’ instead of ‘tenía’ (had).
- Code-switching ($CSE): Words written in Catalan instead of Spanish, e.g., ‘gos’ (‘dog’ in Catalan) instead of ‘perro’ (‘dog’ in Spanish).
- Word omissions ($WOM): Omission of nouns, verbs, articles, prepositions or pronouns that are required to understand the context of the expository text (including argument omissions and subject elisions), e.g., writing “es alto” (‘is tall’) instead of ‘el caballo es alto’ (‘the horse is tall’).
- Functional words errors ($WE): Errors in the use of articles, prepositions or pronouns.
- Errors in nominal inflectional morphemes ($EIM): Changing or omitting a word’s gender inflection (feminine and masculine), e.g., writing ‘el niña’ (‘girl’ with masculine article ‘el’) instead of ‘la niña’ (‘girl’ with feminine article ‘la’); or changing or omitting the nominal number inflection (singular and plural), e.g., ‘los perro’ (singular ‘dog’ with plural article ‘los’) instead of ‘los perros’ (plural ‘dogs’ with plural article ‘los’).
- Verb conjugation errors ($VCE): Verbal inflection errors made when conjugating regular and irregular verbs (errors of number, person or mode). This category also includes errors in gerund and participle use.
- Semantic errors ($SEE): These occur when the child writes one word instead of another, i.e., the child tries to activate a given concept but activates another in the same semantic category (González et al., 2008), e.g., writing ‘gato’ (‘cat’) instead of ‘perro’ (‘dog’), or replacing a word with another semantically unrelated word, e.g., writing ‘yo he abierto la puerta con la bolsa’ (‘I unlocked the door with the bag’) instead of ‘con la llave’ (‘with the key’).
- Pragmatic errors ($PrE): This error is counted when the child uses literal expressions, writes oral expressions or uses a set phrase incorrectly, e.g., ‘El animal te muerde y estás acabado’ (‘The animal bites you and you are done’).

#### 2.3.4 Other

- Language switch ($LS): An occasional use of Catalan to write the text. This category includes switching language for whole sentences, in which case the code is $LSS.
- Colloquialisms ($CW): Slang words, e.g., ‘guay’ (‘cool’) instead of “bueno” (‘good’), ‘mega’ instead of ‘muy’ (‘very’) or ‘chicha’ (colloquial way to refer to ‘meat’ in Spanish) instead of ‘carne’ (meat).

#### 2.3.5 Reliability

Approximately 30% of the written texts (*n* = 16) were randomly selected from the sample to test the reliability using Cohen’s Kappa. Errors were coded by two independent reviewers. The reliability estimates for each writing measure are as follows: MC, 1; CC, 0.93; SC, 0.95; NAj, 0.89; NAv, 0.91; CO, 0.88; SE, 0.97; WOM, 0.92; WE, 0.70; EIM, 1; VCE, 0.82; SEE, 1; PrE, 1; LS, 1; LSS, 1; and CW, 1. If the two evaluators disagreed, they discussed the discrepancy until they reached an agreement. In the exceptional cases that no agreement was reached, the scores of the first author was used in the main analyses.

## 3 Results

### 3.1 Data analysis

Starting with the coding of the expository texts using the CHAT system and the subsequent analysis using CHILDES, we obtained the values of each category for each subject. To assess the differences between the groups, descriptive data for each variable were used, and a non-parametric analysis, specifically the Mann–Whitney U test, was conducted. This choice was made due to the sample size, as it does not follow a normal distribution (as determined by the Shapiro–Wilk test) and the heterogeneity of variances (as determined by Levene’s test). The data is available online in https://n9.cl/0er91.

### 3.2 Word frequency and sentence structure

Table 2 shows the mean, standard deviation and differences between the two groups with respect to word frequency, lexical diversity and sentence structure. The difference between the DLD and TD groups was significant for four out of the seven variables (total number of words, number of different words, total number of clauses and subordinate clauses). Children with DLD wrote significantly fewer words and sentences than TD children. Also, children with DLD wrote texts with less lexical diversity and used a significantly lower proportion of subordinate clauses compared to TD children.

**Table 2:** Mean and standard deviation (SD) of the word frequency and sentence structure variables for the Developmental Language Disorder (DLD) and Typically Developing (TD) groups.

Writing variables	Group					
	DLD (*n = 26*)		TD (*n = 26*)		Comparison	
	Mean	SD	Mean	SD	*z*	*p*
Total number of words	56.54	37.44	131.15	83.281	−3.63	***p* < 0.01**
Number of different words (types/√tokens)	4.78	0.97	6	1.15	−3.56	***p* < 0.01**
Total number of clauses	4.12	1.88	7	3,795	−3.33	***p* < 0.01**
Main clauses (%)	40.94	27.99	30.73	25.15	−1.31	*p* = 0.19
Coordinate clauses (%)	39.43	28.84	29.12	18.54	−1.34	*p* = 0.18
Subordinate clauses (%)	19.63	22.69	40.14	26.75	−2.76	***p* < 0.01**
Words per clause	9.81	3.54	12.53	4.72	−1.95	*p* = 0.051

Significance *p*-values are shown in bold.

### 3.3 Grammatical complexity and lexical density

Table 3 shows the mean, standard deviation and differences between the two groups with regard to grammatical complexity and lexical density. Using the nonparametric Mann–Whitney U test, significant differences between the DLD and TD groups were identified in all three variables. Children with DLD used significantly fewer adjectives, adverbs and connectors compared to TD children of the same age.

**Table 3:** Mean and standard deviation (SD) of the grammatical complexity and lexical density variables for the Developmental Language Disorder (DLD) and Typically Developing (TD) groups.

Writing variables	Group					
	DLD (*n = 26*)		TD (*n = 26*)		Comparison	
	Mean	SD	Mean	SD	*z*	*p*
No. of adjectives	3.92	3.84	10.81	5.46	−2.77	***p* < 0.01**
No. of adverbs	1.69	2.33	7.27	5.65	−4.50	***p* < 0.01**
No. of connectors	3.63	2.73	8.62	7.16	−4.68	***p* < 0.01**

Significance *p*-values are shown in bold.

### 3.4 Errors

**Figure 1:**
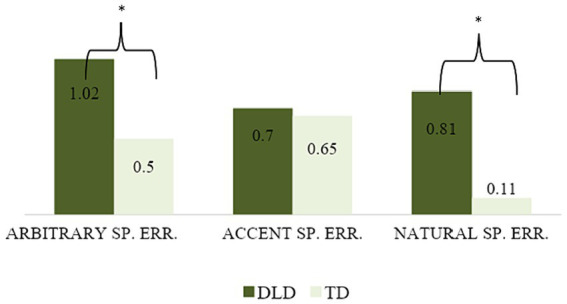
Mean number of arbitrary spelling errors (arbitrary sp. err.), accent mark spelling errors (accent sp. err.) and articulatory spelling errors (natural sp. err) in children with Developmental Language Disorder (DLD) and Typically Developing (TD) children.

Table 4 shows the mean, standard deviation and differences between the two groups with regard to omissions and errors in their expository texts. Significant differences between the two groups were found in 5 out of the 7 variables. The DLD group made significantly more functional words, verb conjugation, nominal inflectional morpheme and spelling errors than the TD group. Children with DLD also omitted more words needed to understand the context of the text. A more detailed analysis was carried out for spelling errors. When the different categories of spelling errors were observed more closely, significant differences appeared between the two groups with respect to arbitrary spelling errors and articulatory spelling errors (z[2.309], *p* < 0.05 and z[3.105], *p* < 0.01, respectively). The children with DLD made significantly more arbitrary spelling errors (mean = 1.02, SD = 0.88) compared to the TD group (mean = 0.5, SD = 0.7). They also made significantly more articulatory spelling errors (mean = 0.81, SD = 1.34) compared to their TD peers (mean = 0.11, SD = 0.21; see Figure 1).

**Table 4:** Mean and standard deviation (SD) of the omissions and errors variables for the Developmental Language Disorder (DLD) and Typically Developing (TD) groups.

Writing variables	Group					
	DLD (*n = 26*)		TD (*n = 26*)		Comparison	
	Mean	SD	Mean	SD	*z*	*p*
Spelling errors	0.20	0.13	0.11	0.09	−3.00	***p* < 0.01**
Word omissions	0.11	0.098	0.031	0.035	−3.97	***p* < 0.01**
Functional words errors	0.03	0.03	0.003	0.007	−3.51	***p* < 0.01**
Errors in nominal inflectional morphemes	0.013	0.017	0.003	0.007	−2.36	***p* < 0.05**
Verb conjugation errors	0.015	0.021	0.001	0.003	−2.78	***p* < 0.01**
Semantic errors	0.005	0.009	0.004	0.009	−0.178	*p* = 0.858
Pragmatic errors	0.007	0.014	0.001	0.005	−0.679	*p* = 0.497

Significance *p*-values are shown in bold.

With regard to switching languages, the results revealed significant differences between the two groups (z[2.194], p < 0.05). The ratio of language switch per word is significantly higher in children with DLD (mean = 0.013, SD = 0.026) compared to TD children (mean = 0.001, SD = 0.003). No significant differences were observed between children with DLD and TD children (z[1.42], *p* = 0.15) in terms of language switches applied to whole sentences. There were also no significant differences between the two groups in the use of colloquialisms (z[0.487], *p* = 0.648).

## 4 Discussion

In this study we explored the Spanish writing abilities of a group of children with DLD in comparison to a group of sex-and age-matched TD children using a written expository text. We were particularly interested in analyzing the writing abilities of this oral-language-impaired population in Spanish. This language is characterized by simple phonological structure, transparent orthography, rich morphology and flexible word order like Spanish.

Building on previous research on English-speaking children with DLD, in this study we analyzed word omissions, inflectional morpheme errors and verb conjugation errors. The analysis showed that the ratio of word omissions, errors in inflectional morphemes marking gender or number, and verb conjugation errors were significantly higher in the texts written by children with DLD compared to TD children of the same chronological age. These results are similar to other studies, such as Mackie et al. (2013), Connelly et al. (2011) and Mackie and Dockrell (2004), who also found that children with DLD had trouble using gender inflectional morphemes, as well as plural (−s), past (−ed) and gerund (−ing) markers, in their writing. Another morphological measurement not reported in previous writing research but key among Spanish-speaking children with DLD are errors using functional words such as articles, prepositions and pronouns that are very frequent in oral language (Bedore and Leonard, 2001; Restrepo and Gutierrez-Clellen, 2001; Sanz-Torrent et al., 2008; Morgan et al., 2013; Coloma et al., 2016). In our study, the children with DLD produced texts with significantly more functional words errors compared to the TD group. All these errors are similar to those made by children with DLD when they express themselves orally, for example difficulties with verb morphology (i.e., No me gusta [:gustan] las avejas [:abejas] / I do not like [number error in Spanish] sheep [: bees]) and in the use of functional words, such as articles (i.e., …la [:las] leonas… / …the [sing.]: the [pl.] lioness…), prepositions (i.e., …pueden oir [:oír] [:a] distansias [:distancias]…/..They can hear [:from] distance) and pronouns (i.e., Mi [:la] raza de mi perro…/ my [:the] breed of my dog.). This clearly shows that such difficulties in oral language production also affect children with DLD’s writing.

Spelling is one of the most impaired aspects of writing among English-speaking children with DLD when compared to TD children (Williams et al., 2013; Dockrell et al., 2014; Reilly et al., 2014). Our results showed that in Spanish, children with DLD also made significantly more spelling errors than their age-matched peers. A more specific analysis showed that they make significantly more articulatory and arbitrary spelling errors. These results are in line with previous research by Mackie and Dockrell (2004), who concluded that most spelling errors are due to letter substitution, insertion or elimination and letter combinations that do not comply with spelling rules. Although the research sample in Mackie and Dockrell (2004) were English-speaking children, their results are similar to ours. This means that children with DLD exhibit difficulties with phonological awareness whether they speak a language with shallow orthography or one with deep orthography.

Children with DLD performed worse in most of the other writing variables compared to their peers in the TD group. These results suggest that children with DLD had more difficulty writing longer texts, i.e., they wrote significantly fewer words and significantly fewer sentences than TD children. Texts by children with DLD were also structurally simpler, contained a significantly lower percentage of subordinate clauses and were not as lexically rich as those written by the TD group. These results track with previous studies done on English-speaking samples (Scott and Windsor, 2000; Puranik et al., 2006; Dockrell et al., 2014; Reilly et al., 2014), where children with DLD produced texts with significantly fewer words and a lower percentage of syntactic units. Our research found no significant differences between the two groups regarding mean sentence length. These results are consistent with Puranik et al. (2006), who found that, despite significant differences between children with DLD and children with TD in sentence production, mean sentence length does not vary significantly between the groups. However, these results contradict research by Mackie et al. (2013) and Scott and Windsor (2000), who found significant differences between the groups, with children with DLD producing fewer words per clause than their TD peers. Regarding clause types, no significant differences were found between the groups in terms of the percentage of simple and coordinate clauses used. There was, however, a difference when comparing the two groups for percentage of subordinate clauses. Proportionally, children with DLD used significantly fewer subordinate clauses than TD children, which indicates that their texts were simpler and less structurally complex. These results were also reported by Mackie and Dockrell (2004).

Along with structural characteristics, this study also evaluated the grammatical richness of the texts. The texts written by the children with DLD contained significantly fewer adjectives, adverbs and connectors (i.e., they were characterized by poor lexical density compared to the same-aged TD children). Another characteristic not analyzed in earlier research related to children with DLD’s writing abilities is the role of pragmatic errors and semantic errors in written texts. We explored these characteristics and did not find significant differences between the DLD and TD groups in these areas.

Finally, we looked for code-switching in the written texts. This measurement was included because the children in our sample were bilingual, speaking both Catalan and Spanish. The results show that the ratio of language switch per word is significantly higher in children with DLD than in TD children, indicating a lack of consistency in language used while writing and supporting the idea that bilingual children with DLD code-switch more than TD children (Pert et al., 2004). This could also be explained as a difficulty in thinking of a word and using the same word in another language as a compensation mechanism. However, as regards oral language, both Gutiérrez-Clellen et al. (2009) and Sanz-Torrent et al. (2007) found that Spanish-English and Spanish-Catalan bilingual children with DLD, respectively, did not differ from age-matched control children in terms of code-switching. Future research might look more closely at code-switching in written texts by bilingual children to analyze the differences between oral and written language.

In summary, writing abilities of children with DLD in Spanish showed more morphology-related, spelling and other writing errors compared to their age-matched TD peers. These results highlight the limitations that children with DLD may face in school when instruction is based on written language, and how these can affect their academic performance.

## 5 Limitations and future directions

This is the first study to explore the characteristics of expository text production in Spanish by children with DLD. However, there are a few areas for future improvement. Although we aimed to recruit as many participants as possible, the final sample consisted of 52 children (26 with DLD and 26 TD children). This sample size aligns with prior studies on English (e.g., Mackie et al., 2013; Dockrell et al., 2014; Andreou and Aslanoglou, 2022; Brimo et al., 2023) and Spanish (Soriano-Ferrer and Contreras-González, 2012; Buil-Legaz et al., 2023) writing abilities in children with DLD. However, our study included participants ranging in age from 7;11 to 15;8 years, representing a diverse range of ages. In order to further enhance the generalizability and reliability of our findings, it is recommended for future studies to expand the sample size and include a more specific age range. By increasing the number of participants and narrowing down the age range, researchers can obtain a more comprehensive understanding of the topic at hand.

Our study centered on the production of expository texts, a common school activity that requires children to plan, translate, and revise. Although we primarily focused on microstructure, the task also allows for macrostructure analysis (global structure and coherence), a crucial factor in gaging the quality of children’s written texts. This aspect could be examined in future studies. The expository text task is less controlled than other tasks like dictation, where evaluators can choose words with different spelling characteristics. When writing expository texts, children can use words they are familiar with, potentially resulting in fewer spelling errors as they may prefer words they can spell correctly. Additionally, even though we allowed unlimited time for children to write a one-page text, the length of their texts significantly varied. Future research should examine the microstructure abilities in children with DLD, and attempt to control the text length to yield a similar amount of information.

In conclusion, the findings from our study should be considered when planning and conducting activities with these children. We emphasize the value of using expository text writing in assessing children with DLD. It is a simple, quick method that yields substantial information about their language and writing skills. Additionally, it would be insightful to examine the effectiveness of interventions targeting oral language issues on improving writing impairments, and vice versa.

## Data availability statement

The datasets presented in this study can be found in online repositories. The names of the repository/repositories and accession number(s) can be found at: https://n9.cl/0er91.

## Ethics statement

The studies involving humans were approved by the Universitat Oberta de Catalunya’s (UOC) Ethics Committee. Furthermore, it was conducted in accordance with the ethical standards laid out in the 1964 Declaration of Helsinki and subsequent updates (WMA. World Medical Association, 2013). The studies were conducted in accordance with the local legislation and institutional requirements. Written informed consent for participation in this study was provided by the participants’ legal guardians/next of kin.

## Author contributions

RB-C: Data curation, Formal analysis, Investigation, Methodology, Validation, Writing – original draft, Writing – review & editing. NA: Data curation, Formal analysis, Investigation, Methodology, Validation, Writing – original draft, Writing – review & editing. MS-T: Formal analysis, Funding acquisition, Investigation, Methodology, Supervision, Writing – original draft, Writing – review & editing. LA: Conceptualization, Formal analysis, Funding acquisition, Investigation, Methodology, Project administration, Supervision, Writing – original draft, Writing – review & editing.
